# Hygral Behavior of Superabsorbent Polymers with Various Particle Sizes and Cross-Linking Densities

**DOI:** 10.3390/polym9110600

**Published:** 2017-11-10

**Authors:** Kyong-Ku Yun, Kwan-Kyu Kim, Wonchang Choi, Jung Heum Yeon

**Affiliations:** 1 Department of Civil Engineering, Kangwon National University, Chuncheon 24341, Korea; kkyun@kangwon.ac.kr; 2 North Gyeonggi Branch, Korea Conformity Laboratories, Pocheon 11184, Korea; kim89@kcl.re.kr; 3 Department of Architectural Engineering, Gachon University, Seongnam 13120, Korea; wchoi@gachon.ac.kr; 4 Department of Civil and Environmental Engineering, Gachon University, Seongnam 13120, Korea

**Keywords:** superabsorbent polymers, particle size, cross-linking density, absorption, desorption, internal curing, mortar, mechanical properties

## Abstract

This study focuses on investigating the effects of particle size and cross-linking density on the hygral behavior of superabsorbent polymers (SAPs), which are increasingly used as an internal curing material for high-performance concrete. Four SAPs with different mean particle diameters and cross-linking densities were tested under controlled wetting and drying conditions to measure free absorption and desorption kinetics. Absorption capacities of SAPs under actual mixing conditions were additionally measured and verified by means of mortar flow and semi-adiabatic hydration heat measurements. In addition, the effects of SAP type and dosage (i.e., 0.2, 0.4, and 0.6% by mass of cement) on the mechanical properties of hardened mortar were assessed. The results indicated that: (1) the absorption capacity increased with decreased cross-linking density and increased particle size under both load-free and mixing conditions; and (2) the greater the cross-linking density and the lower the particle size, the shorter the desorption time. It was also confirmed that while the early-age mechanical properties were more related with the gel strength of swollen SAP, the later-age mechanical properties were more affected by the water retention capacity and spatial distribution of SAP in the matrix.

## 1. Introduction

Superabsorbent polymers (SAPs) are lightly cross-linked polymeric materials that are capable of quickly absorbing a substantial amount of water or aqueous solutions by osmotic pressure [1]. It is well recognized that 1 g of SAP could absorb up to 500 g of aqueous solutions within a few minutes [1,2]. Taking advantage of such super-absorptive characteristics, SAPs have been popularly used in the hygiene, agriculture, forestry, and medical industry since the 1970s [3,4].

In the concrete industry, since Jensen and Hansen [5,6] carried out a pioneering work on the applications of SAPs in cement-based materials (termed “water-entrained cement-based materials”), a number of follow-up studies have been conducted from every corner of the world to seek possibilities to apply SAPs in cement-based materials. Most of the latest studies focused on tackling autogenous shrinkage occurring in high-performance cement-based materials with a low water-to-binder ratio as SAPs can serve as internal reservoirs to supply extra curing water to drying areas (so called “internal curing”), particularly caused by self-desiccation [1,7,8,9,10,11]. Other works performed include the effects of SAP additions on mechanical properties [8,12,13,14,15], rheological properties [16,17,18], self-sealing [19,20,21,22] and self-healing [23], early-age restrained cracking [12,24], creep [25,26], thermal coefficient [27], and freeze–thaw resistance [16,28,29]. The findings were not quite different among the studies: (1) additions of 0.3% to 0.6% SAP by mass of cement were highly effective in mitigating autogenous shrinkage and resulting cracks in cement-based materials, even with and without supplementary cementitious materials (SCMs); (2) SAP additions may adversely affect mechanical properties particularly at early ages; (3) SAP additions increased both yield stress and plastic viscosity of mixtures; (4) SAP effectively blocked cracks upon external water supply, securing watertightness; (5) the restrained stress and stress rate decreased with additions of SAP; (6) the tensile creep of SAP concrete was comparable to that of reference mixture; (7) the “entrained” air voids formed by swollen SAP particles improved the resistance against frost attack; and (8) SAP promoted autogenous healing capacities of cementitious systems. While such works shed some light on multi-functional possibilities of using SAP in cement-based materials, only a few studies are available that have addressed how chemical structures and physical dimensions affect the hygral behavior of SAP [7,30,31,32].

Internal curing efficiency depends on the absorption and desorption kinetics of SAP. If SAP absorbs mixing water quickly, the mixture would lose its workability shortly, which would be harmful to construction quality control. On the contrary, SAP absorbs water slowly or releases absorbed water before setting, a portion of water that acts as mixing water in the cement paste would be greater than designed, consequently leading to degradation of mechanical performance and durability. The desorption rate is also a critical factor that significantly affects internal curing efficiency as it determines the timing and amount of water supply to drying zones [7]. It is well recognized that the absorption and desorption kinetics are closely associated with the cross-linking density, molecular structure, and particle dimensions. This study is intended to provide an in-depth understanding of absorption and desorption kinetics of SAP in terms of particle size and cross-linking density, which would be very useful to enhance knowledge of the internal curing technique using SAPs.

## 2. Materials and Methods

### 2.1. Materials

**Figure 1:**
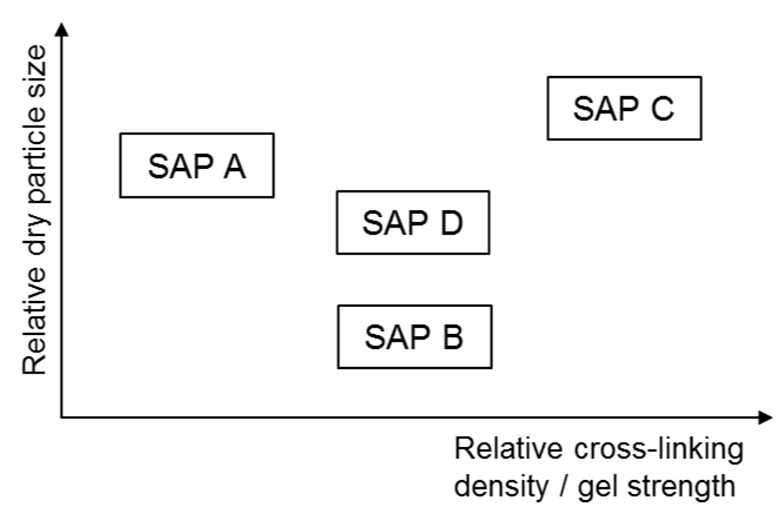
Comparison of cross-linking density/gel strength and particle size among SAPs used (not to scale).

**Figure 2:**
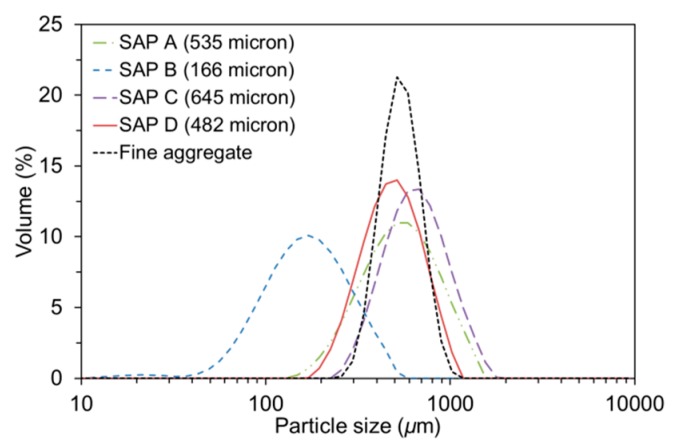
Particle size distributions of SAPs and fine aggregate used.

**Figure 3:**
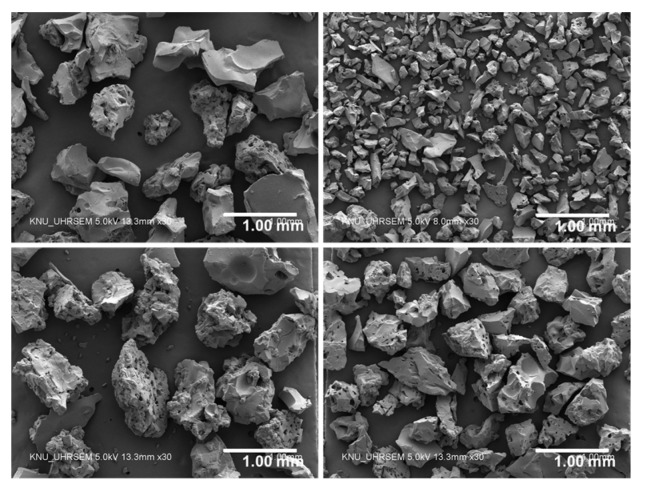
SEM images of SAPs used: SAP A, SAP B, SAP C, and SAP D (clockwise from top left).

This study employed four different types of commercially available SAPs (LG Chem Ltd., Seoul, Korea) made of >92% sodium polyacrylate and <8% water, all of which were produced based on a bulk polymerization technique and then were pulverized into irregular shaped powder. The chemical and physical properties of the SAPs are summarized in Table 1. The SAPs had three different levels of cross-linking density (gel strength) and four different levels of particle size as illustrated in Figure 1. Figure 2 and Figure 3 show the particle size distributions of the dry SAPs and fine aggregate measured using a particle size analyzer (Hydro 2000S; Malvern Instruments, Malvern, UK) and microscopic images of the SAPs captured using an ultra-high resolution scanning electron microscope (UHR-SEM) (Hitachi S-4800; Hitachi, Tokyo, Japan), respectively. The mean dry particle size was 535.01 μm for SAP A, 165.84 μm for SAP B, 644.84 μm for SAP C, and 482.37 μm for SAP D.

Type I portland cement with a specific gravity of 3.15 and a fineness of 3700 cm^2^/g was used. The chemical composition of the cement was: 61.5% CaO, 19.7% SiO_2_, 5.33% Al_2_O_3_, 2.90% Fe_2_O_3_, 3.81% MgO, 2.54% SO_3_, 0.86% K_2_O, and 0.18% Na_2_O.

Standard sand with a specific gravity of 2.65, a fineness modulus of 2.87, an absorption capacity of 1.02%, a SiO_2_ content of 98.4%, and a median diameter (D50) of 0.533 mm was employed as fine aggregate.

### 2.2. Methods

#### 2.2.1. Absorption Test

A “nonwoven bag test” was conducted in accordance with the standard procedure specified in NWSP 240.0.R2 (15) ISO 17190-5 [33] to evaluate the load-free swell capacity of SAPs. The test was initiated with adding 0.20 ± 0.01 g of dry SAP into an empty nonwoven heat-sealed bag with dimensions of 60 × 75 mm^2^, which was then fully soaked in a 25 ± 1 °C solution for prescribed periods of time (i.e., 1, 5, 10, 30, and 60 min). Two solutions were used: distilled water and cement filtrate with a pH of 12.3; the cement filtrate was produced by adding 1 part cement to 10 parts water (*w/c* = 10) and stirring the solution for 12 h using a 5-L Hobart type mixer (HD-111; Hyundai Precision Industry, Co. Ltd., Seoul, Korea) to ensure sufficient dissolution of the cement particles. The revolving speed of the rotary paddle located at the central axis of the mixing bowl was set as 80 rpm. Once the specified time has elapsed, the bag was removed from the solution. Excess solution was immediately dripped using a centrifugal method [32]. Subsequently, the sample was weighted using a digital scale with a readability of 0.01 g (PAG 4201; Ohaus, Parsippany, NJ, USA). Three replicates were tested for each variable. The absorptivity of SAP can be then calculated as

(1) $$AC=\frac{w_{3}-w_{2}-w_{1}}{w_{1}}$$

where *AC* is the absorptivity of SAP (g/g dry SAP); *w*_1_ is the weight of dry SAP (g); *w*_2_ is the weight of a wet bag (g); and *w*_3_ is the weight of a wet bag containing swollen SAP (g).

#### 2.2.2. Desorption Test

To investigate the fundamental desorption behavior of SAPs, a modified desorption test based on [30] was conducted. First, 1 ± 0.1 g of dry SAP was uniformly distributed onto a 3-mm thick acrylic plate with dimensions of 240 × 350 mm^2^, the top side of which was covered with a single layer of double-sided adhesive tapes. Excess SAP particles were removed by turning the plate upside down and softly tapping it. A weighed amount of distilled water determined by means of the abortion test was then sprayed for 1 min to saturate the SAP. After 30 min (at which the SAPs are nearly saturated as will be shown later), excess water stuck between the SAP particles was removed by tilting down the plate about 45° from the horizontal position. The water underneath the plate was wiped with a dry towel. Subsequently, the plate with the saturated SAP was stored in an environmental chamber (simulating a controlled environmental condition of 25 ± 1 °C and 35 ± 1% relative humidity (RH)) until a constant weight was reached while continuously measuring the weight of the SAP using a digital scale (PAG 4201; Ohaus, Parsippany, NJ, USA) that can send the data serially to a computer.

#### 2.2.3. Sorption Isotherm Test

**Figure 4:**
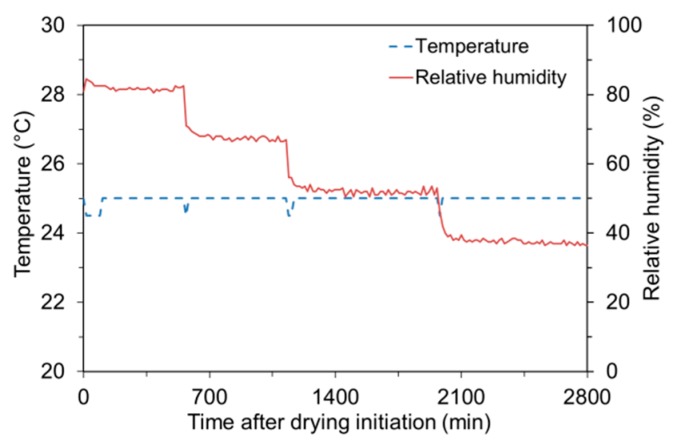
Temperature and RH histories measured during sorption isotherm test.

Since the later-age water migration from SAP to cement paste is primarily governed by the RH gradient between the SAP and cement paste [4,34], it is important to evaluate how the absorptivity varies at different RH levels. SAP samples were prepared with the same procedure utilized in the desorption test to identify the water release across different levels of RH. Absorptivity of the SAP was continuously assessed while varying RH levels inside the chamber from 80% to 35% with a 15% interval as indicated in Figure 4; actual RH levels in the chamber measured using a portable logger (EL-USB-2-LCD; Lascar, Erie, PA, USA) were 82, 68, 53, and 38%. The temperature was kept constant at 25 ± 1 °C. The prescribed RH condition was maintained for a sufficient time period (>8 h) to reach the moisture equilibrium between the SAP and the surroundings. The moisture equilibrium was assumed when the change in weight over any 30-min interval was less than 0.1 g after 8 h of drying initiation under the specified RH level.

#### 2.2.4. Absorption Test under Mixing Conditions

To evaluate the absorption capacity of SAPs under actual mixing conditions with the presence of multispecies ions (due to dissolution of cement particles) and external pressure, flow of fresh mortar mixtures incorporating different amounts of SAP was measured as per ASTM C1437 (“Standard Test Method for Flow of Hydraulic Cement Mortar”). The ideas behind of this approach are: (1) the flow solely relies on the free water available after water uptake of SAP as long as the basic mixture proportions are identical; and (2) the hydrogel has a minimal contribution to the shear flow behavior of a fresh mixture [30]. The absorptivity resulting in the same flow with control mixture was defined as the actual absorption capacity in fresh mortar. Furthermore, the flow values of six trial mixtures whose effective *w/c* was adjusted per the predetermined absorption capacities were measured to verify the absorption capacity measured.

Table 2 summarizes the mixture proportions used in determination of absorptivity in fresh SAP mortar, along with those used in verification tests. The effective *w*/*c* and fine aggregate-to-cement ratio for absorptivity tests were fixed as 0.485 and 2.75, respectively. For verification mixtures, two levels of *w/c* (i.e., 0.485 and 0.30) were tested. The fine aggregate-to-cement ratios for each *w/c* were 2.75 and 2.1, respectively. The volume of internal curing water was assumed to be negligible because the amount of SAP added was quite small and the internal curing water would bind chemically with cement shortly upon release. The mixing was done using a Hobart-type mixer with the following sequences:

- Dry mix all the dry ingredients (cement, sand, and dry SAP) for 1 min with 140 rpm.
- Add water for 30 s with a mixing intensity of 140 rpm.
- Mix for 1 min with a mixing intensity of 285 rpm.
- Scrap mortar from the walls and manual mix for 1 min.
- Final mix for 1 min with a mixing intensity of 285 rpm.

#### 2.2.5. Semi-Adiabatic Temperature Rise Test

A custom-built semi-adiabatic calorimeter was employed to validate the measured absorption capacity using early-age hydration heat evolution data. This method is based on the premise that the early-age hydration depends on the remaining water in the cement paste after SAP absorbs part of mixing water while the inclusion itself hardly affects the rate of hydration heat evolution [35]. Thus, if the SAP mixture has the same “effective *w/c*” as the control mixture, both mixtures would have similar semi-adiabatic hydration heat profiles. 

The semi-adiabatic calorimeter is a cubical box with inner dimensions of 20 × 20 × 20 cm^3^. As the wall material, 20-cm thick expanded polystyrene foam panels were used due to its high insulation efficiency. To prevent the water leakage and heat loss through the joints when a sample is poured, insulation tape was applied along the all joints of the calorimeter. Temperature evolutions at the center of the specimen were measured every 10 min using a K-type thermocouple until the specimen temperature converges to the ambient temperature of 20 °C. All the materials were stabilized at 20 ± 1 °C for 24 h prior to mixing to minimize the effect of materials’ initial temperature.

#### 2.2.6. Flexural, Compressive Strength, and Elastic Modulus Tests

Flexural strength was measured in accordance with ASTM C348 (“Standard Test Method for Flexural Strength of Hydraulic-Cement Mortars”). Specimens were cured under 23 ± 1 °C and 95% RH for 24 h of casting and then stored in double layers of air-tight plastic bags to ensure moisture retention. Three 40 × 40 × 160 mm^3^ replicate specimens were tested by center point loading with a controlled load rate of 50 N/s using a 200-kN universal testing machine. 

Compressive strength was measured as per ASTM C349 (“Standard Method for Compressive Strength of Hydraulic-Cement Mortars Using Portions of Prisms Broken in Flexure”). A total of six specimens were tested for each variable with a controlled load rate of 2400 N/s using the same unit used in the flexural test.

Modulus of elasticity was measured using three parallel specimens with dimensions of ϕ 50 × 100 mm following the standard procedure in ASTM C469 (“Standard Test Method for Static Modulus of Elasticity and Poisson’s Ratio of Concrete in Compression”). To measure the compressive strain upon load application, two electrical resistance strain gages (Tokyo Sokki; PFL-10-11-3L) were attached longitudinally on the surface of the specimen.

The mixture proportions used in the mechanical tests were determined considering actual moisture uptake capacity under mixing conditions as shown in Table 3. To identify the pure effect of entrained pores formed by the hydrogel on the mechanical properties (with minimal contributions of internal curing), a high effective *w*/*c* of 0.485 was selected.

## 3. Results and Discussion

### 3.1. Absorption Kinetics

**Figure 5:**
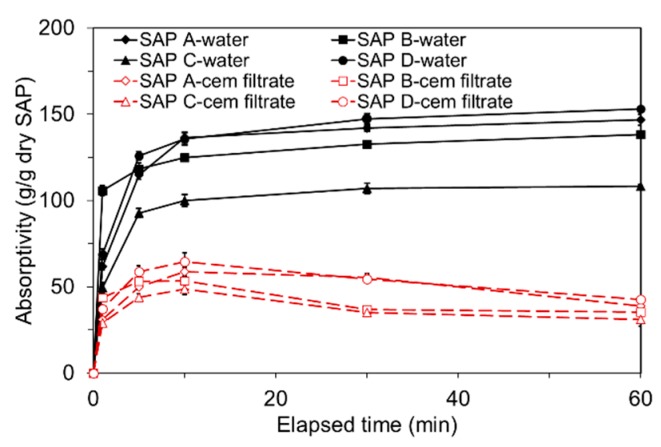
Absorption kinetics of various SAPs in distilled water and cement filtrate.

The absorption responses of SAPs exposed to distilled water and cement filtrate are presented in Figure 5. First, it is noted that the absorptivity measured in distilled water and cement filtrate showed a different behavior. The absorptivity measured in cement filtrate was less than a half of that measured in distilled water because the alkaline environment of the cement solution (pH 12.3) reduced the osmotic pressure that drives fluid uptake of SAP [4,7,32]. For both distilled water and cement filtrate, an abrupt increase in absorptivity was observed within the first 10 min of soaking. However, while the absorptivity measured in distilled water converged to an almost constant value after 10 min due to the elastic retraction forces acting on the cross-linked polymer chains [30], the absorptivity measured in cement filtrate gradually decreased over time. This is due to the neutralization of SAP resulting from the interaction between anionic carboxylate groups of the polymer and Ca^2+^ ions presenting in the solution, which uncharged the polymer chains [30,32]. 

The initial absorption rate was greatest for SAP B in both distilled water and cement filtrate due to its finest particle size and resulting larger specific surface area; SAP B reached 84.8% and 81.5% of the 10-min absorptivity in distilled water and cement filtrate, respectively, only within the first minute of soaking. However, the 60-min absorptivity of SAP B was rather slightly lower than that of SAP A and SAP D having a lower cross-linking density and a greater particle size, respectively, which was consistent with the findings of previous investigations [7]. The findings led to the following conclusions: (1) the absorption rate increases with the lower particle diameter; and (2) the absorption capacity increases with decreased cross-linking density and increased particle diameter. 

### 3.2. Desorption Kinetics

**Figure 6:**
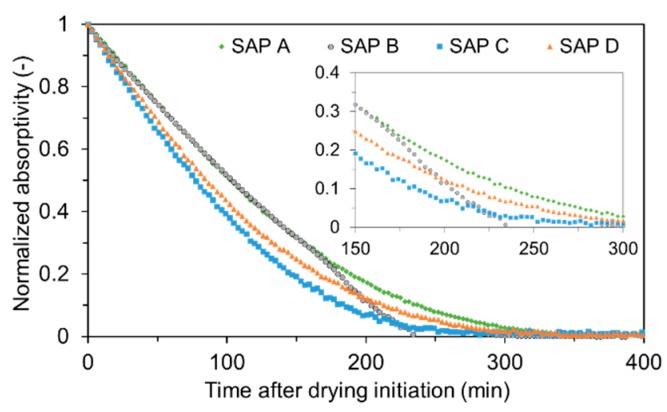
Desorption kinetics of SAPs.

The desorption behavior of SAPs is presented in Figure 6, the *y*-axis of which denotes the normalized weight calculated based on the weight of water initially absorbed in SAP and the weight of remaining water during the drying process. Note that the saturated SAPs exhibited almost linear weight losses for the first 100 min, and then the rate noticeably decreased thereafter, except for SAP B. This is because the surface water in SAP can be readily emitted upon drying since it is more subjected to less active and weak van der Waals forces, while the water closer to the core is further held by strong hydrogen bonds formed between the side chains of the polymer [6,30,31]. This effect delayed the diffusion of the core water to the drying front, which consequently led to nonlinear drying curves. For SAP B, however, it should be noted that the desorption curve remained almost linear over the whole drying period unlike other SAPs. This is possibly because the water absorbed in SAP B is mostly surface water due to its smaller particle diameter, which is less hindered by hydrogen bonds compared to other types of SAP [30,31].

The desorption data also shows that SAP C (highest cross-linking) emptied in 5 h after drying initiation whereas SAP A (lowest cross-linking) reached complete drying in about 6 h. One possible reason for this behavior is that the more the cross-links between polymer chains there were, the more elastic retraction forces acted in the network structure [7], which “squeezes “ out the absorbed water in SAP. In addition, even though SAP B (smallest particle size) and SAP D (largest particle size) had an identical cross-linking density, there was a significant discrepancy in drying time. The reasons for the shorter drying time of SAP B are: (1) the smaller particles have larger exposure areas and a small nucleus and thus can release more water for a given drying period [31]; and (2) smaller particles are more exposed to weaker intermolecular attraction forces (i.e., van der Waals forces) because of their larger surface zone as compared to larger ones [31]. 

### 3.3. Sorption Isotherm

**Figure 7:**
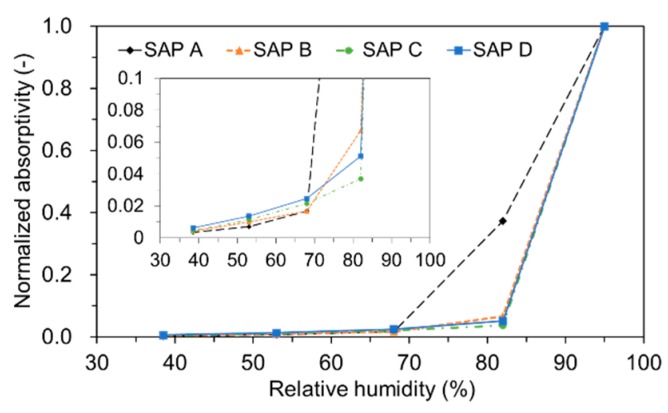
Sorption isotherm of SAPs.

Figure 7 depicts the relationships between the normalized absorptivity and RH measured for each SAP. Here, the maximum RH was all assumed to be 95% as an air medium cannot practically guarantee full saturation. Similarly with [6], most of the absorbed water was released above 85 to 90% RH for all the SAPs, indicating that the SAPs can provide good internal curing efficiency particularly for cement-based materials with a low *w/c* [36,37]. 

It is also interesting to note that even though SAP B, SAP C, and SAP D all showed a quite similar sorption isotherm across the measured RH levels, SAP A retained approximately seven times more water at 82% RH. It appears that smaller cross-linking density and larger particle size of SAP A contributed the greater moisture retaining capacity as the larger-sized and less cross-linked SAP’s network structure provides more spaces that water molecules can be captured by stronger binding forces (i.e., hydrogen bonds) [6,7,30]. This may provide inferences that SAP A has a potential to be used as an internal curing material in applications where a prolonged curing period is required.

### 3.4. Absorption Capacity in Fresh Mortar

**Figure 8:**
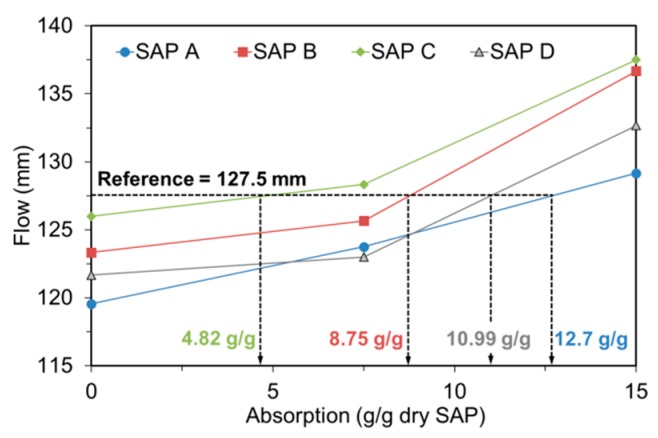
Determination of absorption capacity of SAPs under mixing conditions.

Figure 8 displays how the absorption capacity of SAPs was estimated under mixing conditions. The *x*-axis of each figure is the assumed absorptivity of SAP, and the *y*-axis denotes the spread diameter of fresh mortar measured immediately after mixing. The results indicate that the higher the assumed absorptivity, the greater the flow. This is because as the assumed absorptivity becomes greater, the effective water content that serves as mixing water increases. Once the variation of the flow was measured for each SAP, the absorptivity resulting in same flow with control mixture (127.5 mm) was back-calculated using linear interpolation. The logic behind this analysis method is that if the absorption capacity is overestimated or underestimated than actual, the flow would be greater or less than that of control mixture. The measured absorptivity was greatest for SAP A (12.70 g/g dry SAP) and smallest for SAP C (4.82 g/g dry SAP). The finding is in good agreement with those from previous studies [4,7] in that the absorptivity decreases as the cross-linking density increases. In addition, about a 20% difference in absorptivity was noted between SAP B and SAP D, which demonstrates that the absorptivity of SAP relies on not only the cross-linking density but also the particle size.

**Figure 9:**
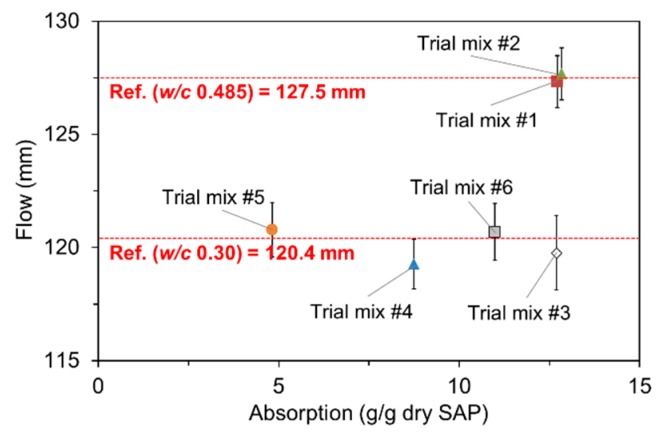
Verification of measured absorption capacity using trial mixtures.

To verify the effectiveness of the method used, six trial mixtures (two with an effective *w/c* of 0.485 and four with an effective *w/c* of 0.30) were additionally tested. Two mixtures with the effective *w/c* of 0.485 were prepared with 0.2% and 0.4% SAP A by mass of cement, whose water content was adjusted per the measured absorptivity (i.e., 12.70 g/g dry SAP) that leads to the effective *w/c* of 0.485. The rest of the mixtures were prepared using four different types of SAPs with a fixed dosage of 0.4% by mass of cement. The measured absorptivities indicated in Figure 8 were used to obtain the target effective *w/c* of 0.30. As seen in Figure 9, all the trial mixtures yielded almost similar flow values with control mixtures, regardless of the *w*/*c* and the type and dosage of SAP, indicating that the method was quite effective in evaluating the actual absorptivity of SAP under realistic mixing conditions.

### 3.5. Semi-Adiabatic Temperature Rise

**Figure 10:**
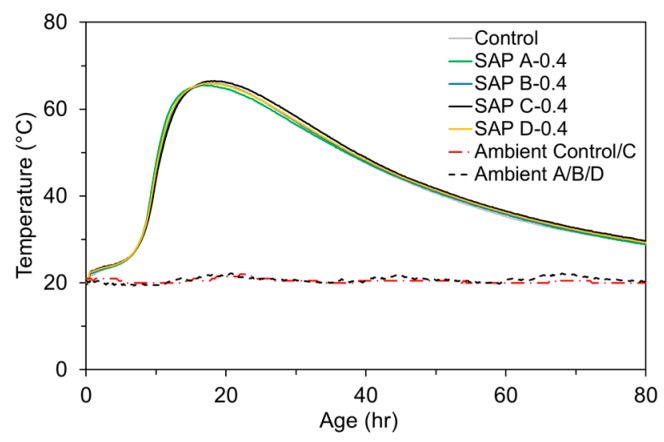
Semi-adiabatic temperature rise for control and 0.4% SAP mixtures.

The semi-adiabatic temperature evolutions for mixtures with 0.4% SAPs and without SAP are compared in Figure 10. The water content for all the SAP mixtures was adjusted per the measured absorption capacities to achieve the consistent effective *w*/*c* of 0.485. The semi-adiabatic hydration heat measurements confirm that the early-age heat evolution was directly associated with the “effective *w/c*” as the hydration heat evolution of all the SAP mixtures was quite close to that of control mixture, particularly at the ascending branch (<15 h). At later ages (>40 h), the temperature was slightly higher for the SAP mixtures than the control mixture, which seems to be attributed to the enhanced degree of hydration by internal curing effects. The slightly lower temperature profile of the SAP A mixture between 15 and 40 h appears to be associated with its slower desorption kinetics.

### 3.6. Mechanical Properties

**Figure 11:**
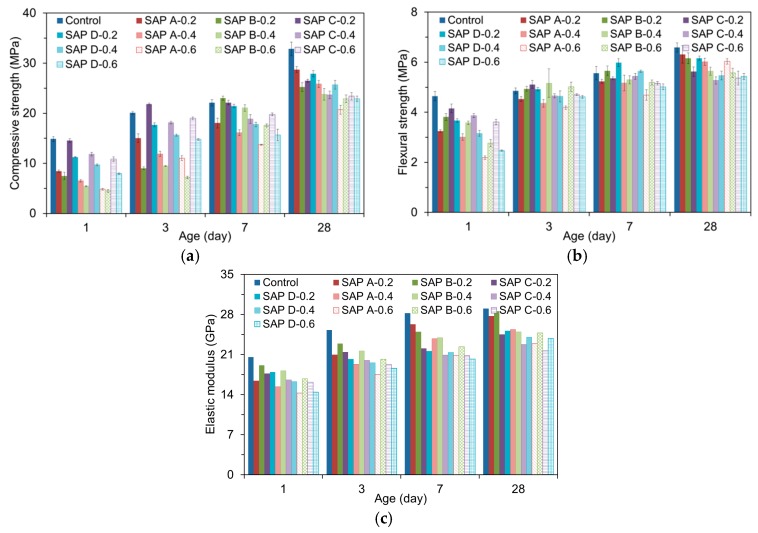
Mechanical properties of SAP mortars: (**a**) compressive strength; (**b**) flexural strength; (**c**) modulus of elasticity.

The influences of SAP type and dosage on the mechanical properties are illustrated in Figure 11. Similarly with previous investigations [8,12,13,14,15,38], most of the SAP mixtures underwent notable reductions in mechanical properties compared to the reference mixture. The mechanical performance reduction was greater as the amount of SAP addition increased, even though all the samples had the same effective *w/c* of 0.485. This is because (1) the swollen SAPs form numerous macropores in the matrix as they lose absorbed water, increasing capillary porosity [15,29,34,39]; (2) the hydrogel (swollen SAP) has a much lower stiffness than the hydrated matrices [2]; (3) some macropores served as stress risers due to their irregular geometry [15]; and (4) the increased SAP addition delayed the setting time [15]. In particular, it is interesting to note that the compressive strength was much more affected by the SAP additions. When the SAP was added by 0.2, 0.4, and 0.6% by mass of cement, the 28-day compressive strength was reduced by 17.6, 29.7, and 36.2%, respectively, while the 28-day flexural strength was reduced by 8.1, 15.1, and 15.0%, respectively, compared to the reference mixture. This is most probably because the SAPs formed numerous irregular-shaped macropores in the matrix that are not capable of carrying the compression by dome action [15], which more affected the compression behavior than flexural behavior. The reduction rates of elastic modulus with SAP additions were found to be quite similar to those of flexural strength (i.e., 8.8, 16.4, and 19.7% with 0.2, 0.4, and 0.6% SAP additions, respectively) since the specimens for elastic modulus measurement were not subjected to a load level as high as to give rise to failure in compression.

On the other hand, the relatively higher mechanical properties of SAP C mixtures at early ages indicate that the early-age mechanical properties may be associated with the gel strength of swollen SAP. This is because, at early ages, the SAP is not fully detached from the pore walls due to the smaller RH gradient between the SAP and pore walls and thus partly contributes to a load bearing capacity while maintaining geometrically stable shapes [2]. Plus, the faster water release of SAP C compared to other SAPs appeared to promote the early-age degree of hydration, and in turn, the early-age strength gains. However, the later-age mechanical properties were rather higher for SAP A mixtures than other SAP mixtures. The result is attributed to the better moisture retention performance of SAP A as it can supply internal curing water for a longer period, contributing to the later-age strength gains. SAP B mixtures also showed enhanced mechanical properties at later ages, which can be explained by the improved internal curing efficiency due to the wider spatial distribution of finer SAP B [9].

## 4. Conclusions

This study presents how the particle size and cross-linking density affect the absorption and desorption kinetics of superabsorbent polymers (SAPs), one of the promising internal curing additives for high-performance concrete. In addition, the effects of particle size and cross-linking density on the mechanical properties of cement mortar were evaluated. To identify the individual impacts of SAP and the pores provided by swollen SAPs with a minimal contribution of internal curing effect, a standard water-to-cement ratio (*w/c*) of 0.485 was chosen for the mechanical tests. The key findings can be drawn as follows:

- While the free absorptivity measured in distilled water reached a nearly constant level that measured in high-ionic cement filtrate gradually decreased due to the neutralization of SAP.
- The absorption capacity of SAP under both load-free and actual mixing conditions increased with decreased cross-linking density and increased particle size. The absorption rate was greater for finer SAP.
- The greater the cross-linking density of SAP, the shorter the desorption time. The rate of water release was higher for finer SAP due to weaker intermolecular attraction forces.
- The sorption isotherm showed that most of the absorbed water was released above 85 to 90% RH for all SAPs, demonstrating a potential benefit of SAPs in internal curing.
- The flow- and hydration heat-based methods were found to be feasible to evaluate the actual absorptivity of SAP under mixing conditions.
- SAP additions overall reduced the mechanical properties of mortar. While the flexural strength and elastic modulus were reduced by about 8 to 20% compared to the reference mixture, the compressive strength was reduced by about 18 to 36%. This indicates that the SAP additions more affected the compressive strength than flexural strength and elastic modulus.
- Whereas the early-age mechanical properties were more related with the gel strength of swollen SAP particulates, the later-age mechanical properties were more concerned with the moisture retention capacity and spatial distribution of SAP.

Because the primary merit of SAPs in cement-based materials is in their shrinkage mitigation capabilities, especially at early ages, a future research effort is needed to characterize how the particle size and cross-linking density of SAPs are related to autogenous shrinkage and resulting restrained stress developments.

## Figures and Tables

**Table 1:** Chemical and physical properties of SAPs used.

Chemical Nomenclature	Chemical Formula	Molar Mass (g/mol)	Constitutional Formula	Density (g/cm^3^)	Appearance
Poly (sodium prop-2-enoate)	(C_3_H_3_NaO_2_)_n_	Variable	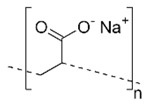	1.22	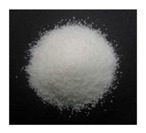

**Table 2:** Mixture proportions of SAP mortar used to determine and verify absorptivity in fresh mortar.

Material	Weight Per Unit Volume (kg/m^3^)										
	Control 0.485	Control 0.30	AC ^a^ 0 g/g	AC 7.5 g/g	AC 15 g/g	Trial Mix 1 (SAP A 0.2%)	Trial Mix 2 (SAP A 0.4%)	Trial Mix 3 (SAP A 0.4%)	Trial Mix 4 (SAP B 0.4%)	Trial Mix 5 (SAP C 0.4%)	Trial Mix 6 (SAP D 0.4%)
Cement	527.1	696.5	527.1	527.1	527.1	527.1	527.1	696.5	696.5	696.5	696.5
Water	255.6	209.0	255.6	263.5	271.4	269.0	282.4	244.3	233.3	222.4	239.6
Sand	1449.5	1462.7	1449.5	1449.5	1449.5	1449.5	1449.5	1462.7	1462.7	1462.7	1462.7
SAP	0	0	1.054	1.054	1.054	1.054	2.108	2.786	2.786	2.786	2.786

^a^ Assumed absorption capacity.

**Table 3:** Mixture proportions of SAP mortar used for mechanical tests.

Specimen	Weight Per Unit Volume (kg/m^3^)					Total *w/c* ^b^
	Cement	Water	Sand	SAP	IC Water ^a^	
Control	527.1	255.6	1449.5	0	0	0.485
SAP A-0.2	519.9	252.2	1429.7	1.04	13.21	0.510
SAP A-0.4	512.9	248.8	1410.5	2.05	26.06	0.536
SAP A-0.6	506.1	245.5	1391.8	3.04	38.56	0.561
SAP B-0.2	522.1	253.2	1449.5	1.04	9.14	0.503
SAP B-0.4	517.3	250.9	1422.6	2.07	18.11	0.520
SAP B-0.6	512.5	248.6	1409.4	3.08	26.91	0.538
SAP C-0.2	524.3	254.3	1441.8	1.05	5.05	0.495
SAP C-0.4	521.6	253.0	1434.4	2.09	10.06	0.504
SAP C-0.6	518.9	251.7	1427.0	3.11	15.01	0.514
SAP D-0.2	520.9	252.6	1432.5	1.04	11.45	0.507
SAP D-0.4	514.8	249.7	1415.7	2.06	22.63	0.529
SAP D-0.6	508.9	246.8	1399.5	3.05	33.56	0.551

^a^ Internal curing water: extra water initially stored in SAP; ^b^ Theoretical *w*/*c* determined based on both mixing water and internal curing water.
